# A Re-Evaluation of the Utility of Symptom Checklist-90-Revised for Measuring the Spectra in the Hierarchical Taxonomy of Psychopathology

**DOI:** 10.3390/pediatric16040093

**Published:** 2024-12-09

**Authors:** Rapson Gomez, Daniel Zarate, Taylor Brown, Vasileios Stavropoulos

**Affiliations:** Psychology Department, School of Health and Biomedicine, Bundoora Campus, RMIT University, Melbourne, VIC 3000, Australia; rapson.gomez@federation.edu.au (R.G.);

**Keywords:** adolescents, Greece, Hierarchical Taxonomy of Psychopathology (HiTOP), Symptom Checklist-90-Revised (SCL-90-R), Big-Five personality dimensions

## Abstract

The present study examines the potential of the Symptom Checklist-90-Revised (SCL-90-R) as a measure for the Hierarchical Taxonomy of Psychopathology (HiTop) model. Two structural models were evaluated. In Model 1, the SCL-90-R dimensions were allocated to somatoform (comprising somatization), internalizing (comprising obsessive–compulsive, interpersonal sensitivity, depression, anxiety, and phobic anxiety), and antagonistic disinhibited (comprising hostility) spectra. Model 2 included an additional detachment spectrum (comprising paranoid ideation and psychoticism). Method: A total of 1594 adolescents [52.2% boys; age ranged from 14 to 17 years; mean age (*SD*) = 16.04 years (0.737 years)] from the general community in Athens completed the SCL-90-R and the Funf-Faktoren-Fragebogen fur Kinder (FFFK). Confirmatory factor analysis (CFA) was conducted to validate the proposed models. Results: The findings supported Model 1, demonstrating adequate global fit, salient and significant factor loadings, discriminant validity, reliability, and external validity of the factors. Conclusions: These results indicate that the SCL-90-R scales of somatization, obsessive–compulsive, interpersonal sensitivity, depression, anxiety, hostility, and phobic anxiety are appropriate measures for the corresponding HiTop dimensions. However, the scales for paranoid ideation and psychoticism were not suitable for this purpose. The theoretical contributions and conclusions are discussed, highlighting the implications of these findings for the clinical and theoretical application of the SCL-90-R in psychopathological assessment and research.

## 1. Introduction

The Symptom Checklist-90-Revised (SCL-90-R; [1,2]) is a widely used self-report questionnaire designed to measure a range of psychological and psychiatric symptoms. The SCL-90-R symptoms encompass somatization (distress related to bodily or physiological experiences), obsessive–compulsive (intrusive thoughts and compulsive actions), interpersonal sensitivity (self-perceived inadequacy or inferiority in relationships), depression (low mood and decreased sense of meaning), anxiety (anxious symptoms and experienced tensions), hostility (aggressiveness towards others), phobic anxiety (fears related to specific stimuli), paranoid ideation (projections onto others and persecutory cognitions), and psychoticism (psychotic and schizophrenic behaviours).

In a recent study involving adolescents, Gomez et al. [3] examined the utility of the SCL-90-R dimensions for assessing constructs in the Hierarchical Taxonomy of Psychopathology (HiTOP) model. HiTOP is a recently proposed dimensional model of psychopathology that aims to provide a more comprehensive understanding of the organization of mental disorders [4]. Gomez et al. [3] found that the SCL-90-R dimensions of somatization, obsessive–compulsive, interpersonal sensitivity, depression, anxiety, hostility, and phobic anxiety were associated with the Big-Five personality dimensions [5,6,7] as theoretically predicated by the HiTOP model. However, the dimensions for paranoid ideation and psychoticism did not show similar associations.

Based on these findings, the authors concluded that the SCL-90-R scales for somatization, obsessive–compulsive tendencies, interpersonal sensitivity, depression, anxiety, hostility, and phobic anxiety can be effectively used to measure the corresponding dimensions (subfactors) in the HiTOP model, as well as the associated spectra and super spectra. Despite these conclusions, we argue that the recommendation by Gomez et al. [3] may be premature. In this study, we propose an alternative perspective, suggesting that all nine SCL-90-R dimensions, including paranoid ideation and psychoticism, have the potential to measure the HiTOP subfactors and their associated spectra and super spectra.

In the Gomez et al. [3] study, the authors mapped the SCL-90-R dimensions onto the associated spectra and super spectra of the HiTOP model (see Supplementary Table S1). Specifically, the SCL-90 somatoform dimension aligned with the somatoform spectrum, which falls under the emotional dysfunction super spectrum. Depression, anxiety, interpersonal sensitivity, obsessive–compulsive, and phobic anxiety were considered part of the internalizing spectrum, also within the emotional dysfunction super spectrum. Hostility was categorized under the antisocial subfactor, fitting into the agonistic disinhibited spectrum and the externalizing super spectrum. Paranoid ideation and psychoticism were classified as thought disorder subfactors, components of the thought disorder spectrum, and part of the psychosis super spectrum factor.

The HiTop model is suggested to be underpinned by the maladaptive variants of the extensively validated and replicated Five-Factor model (FFM) of personality, also known as the Big Five [8]. The FFM [5,6,7] contains broad personality dimensions, including extraversion (individual differences in reactivity to positive environmental stimuli), neuroticism (differences in reactivity to negative environmental stimuli) or emotional stability, agreeableness (differences in maintaining social harmony), conscientiousness (differences in organization, responsibility, and task-focus), and openness to experience (differences in being “open-minded”). According to the HiTop model, the equivalent maladaptive variants of personality provided in Section III of DSM-5 [8] are negative affectivity, detachment, antagonism, disinhibition, and psychoticism, respectively [9].

Considering this, Gomez et al. [3] speculated that the SCL-90-R dimensions of depression, anxiety, interpersonal sensitivity, obsessive–compulsive, and phobic anxiety, which collectively reflect the HiTop internalizing spectrum, would be negatively associated with Big-Five emotional stability. Additionally, it was hypothesized that the SCL-90-R dimension of hostility, part of the HiTop antagonistic disinhibited spectrum, would be negatively associated with Big-Five agreeableness. While paranoid ideation and psychoticism, reflecting the HiTop thought disorder spectrum, were expected to be positively associated with Big-Five openness to experience. Finally, the SCL-90-R somatization, part of the HiTop somatoform spectrum, was predicted to be negatively associated with the Big-Five emotional stability.

The findings from Gomez et al. [3] showed that the SCL-90-R dimensions of somatization, obsessive–compulsive, interpersonal sensitivity, depression, anxiety, hostility, and phobic anxiety were indeed associated with the Big-Five personality factors as speculated. However, the SCL-90-R dimensions for paranoid ideation and psychoticism did not show the predicted associations. Based primarily on these associations with the Big-Five personality dimensions, the authors interpreted the findings as providing reasonable support for the use of the SCL-90-R scales of somatization, obsessive–compulsive, interpersonal sensitivity, depression, anxiety, hostility, and phobic anxiety within the HiTop model. Conversely, since paranoid ideation and psychoticism were not associated with openness as predicted by the HiTop model, the authors concluded that these scales are not useful for measuring HiTop constructs.

Although Gomez et al. [3] have recommended the omission of the paranoid ideation and psychoticism when examining the HiTop model, this recommendation may be premature. Their recommendation was based on their findings that both paranoid ideation and psychoticism were not associated with the Big-Five openness dimensions, as could be predicted from the HiTop model. Indeed, other past studies have also shown no associations between psychoticism with openness (e.g., [3,10,11,12], and paranoid ideation with openness (e.g., [3,10,12]. One exception is that Schmitz et al. [11] found a significant positive correlation between paranoid ideation and openness. Additionally, existing data do not support the view that psychoticism in the dimensional trait model of psychopathology is equivalent to openness in normal personality [13,14,15]. Thus, the assumption that paranoid ideation and psychoticism would be associated with openness may be flawed. An updated version of the personality dimensions underlying the HiTOP model has highlighted the weak and inconsistent associations between psychoticism and openness [9].

As for the associations of paranoid ideation and psychoticism with the Big-Five personality dimensions, past studies have generally reported significant negative correlations for both paranoid ideation and psychoticism with emotional stability, extraversion, and conscientiousness [11,12]. They have also shown negative, but relatively low, association with agreeableness [11]. Smith and Snell [16] reported significant negative partial correlations for paranoid ideation (but not psychoticism) with emotional stability, extraversion, and conscientiousness. Brewer [10] reported unique negative associations of paranoid ideation and psychoticism with emotional stability. Given these findings, it could be speculated that SCL-90-R paranoid ideation and psychoticism are more likely to be linked to emotional stability and extraversion than the other Big-Five personality dimensions. Considering this, it could be further speculated that paranoid ideation and psychoticism could be components of the detachment spectra and psychosis super spectra, which have been hypothesized to be associated negatively with extraversion.

**Figure 1:**
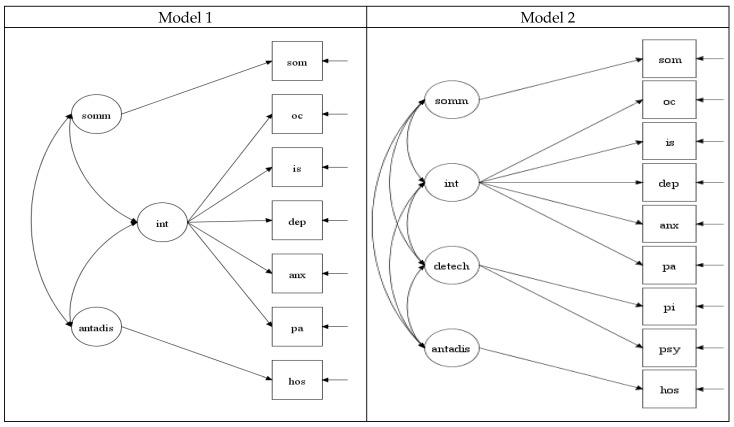
Standardized factor loading and correlations of latent factors for Model 1 and Model 2. som = somatization; oc = obsessive–compulsive; is = interpersonal sensitivity; dep = depression; anx = anxiety; hos = hostility; pa = phobic anxiety; pi = paranoid ideation; psy = psychoticism; int = internalizing; antadis = antagonistic disinhibited; detach = detachment; somm = somatoform Model 1 that excluded the SCL-90-R paranoid ideation and psychoticism dimensions had latent factors for internalizing, antagonistic disinhibited, and somatoform. Model 2 that included the SCL-90-R paranoid ideation and psychoticism dimensions had latent factors for internalizing, antagonistic disinhibited, somatoform, and detachment.

In summary, related to the HiTop model at the level of the spectra, the present study proposed two possible configurations (see Figure 1). In line with past research (i.e., [3]), one model (referred to as Model 1) includes seven of the SCL-90-R dimensions (excluding paranoid ideation and psychoticism dimensions), where SCL-90-R somatization is in the somatoform spectrum; SCL-90-R obsessive–compulsive, interpersonal sensitivity, depression, anxiety, and phobic anxiety dimensions are in the internalizing spectrum; and SCL-90-R hostility is in the antagonistic disinhibited spectrum. The other model (referred to as Model 2) includes all nine SCL-90-R dimensions. In this model, SCL-90-R somatization is in the HiTop somatoform spectrum; SCL-90-R obsessive–compulsive, interpersonal sensitivity, depression, anxiety, and phobic anxiety are in the HiTop internalizing spectrum; SCL-90-R hostility is in the HiTop antagonistic disinhibited spectrum; and paranoid ideation and psychoticism are in the HiTop detachment spectrum. To date, the relative support for these models has not been tested and compared and, therefore, requires further attention. As will be noticed, although there are data linking paranoid ideation and psychoticism with agreeableness, we did not consider a model with these links as the findings for these links have been relatively low [11].

## 2. Aim of Study

Following the literature presented above, the aim of the current study was to test the support for structural Model 1 (the initial model proposed by Gomez et al. [3] and structural Model 2 (alternate model with all nine SCL-90 dimensions). Unlike the previous study by Gomez et al. [3], which involved Greek adolescents from Cyprus, the current study focused on Greek adolescents from Athens. The preferred model was selected on the basis of global fit, including salience and significant factor loadings, and the discriminant validity of the latent factors. Additionally, the internal consistency reliability coefficients of the latent factors were assessed, as well as the external validity, determined by the associations with the Big-Five personality dimensions as predicted by the HiTop model.

## 3. Method

### 3.1. Participants

The initial sample involved 2090 students at public Greek high schools in the broader Athens metropolitan area. Only the participants who completed ratings for all study measures were involved in this study. The final sample involved 1594 students. There were 832 (52.2%) boys and 732 (47.8%) girls. Their age ranged between 14 and 17 years, with a mean of 16.04 years (*SD* = 0.737 years). The mean (*SD*) age for boys and girls were 16.08 years (0.743 years) and 16.01 years (0.30 years), respectively. Boys and girls did not differ significantly in age, *t* = 1.180, *p* < 0.062. In relation to residence, data were available for 1594 participants. In all, 1477 (92.7%) were residing with parents and siblings, 10 (0.6%) with grandparents, 15 (0.9%) with parents, siblings and grandparents, and 89 (5.6%) identified “others” category. For the highest educational level, both parents completed at least high school (>90%).

### 3.2. Measures

As part of this study, parents of participants provided information on the age and sex of their adolescent targeted for this study. They also provided information on their own marital and educational status. All adolescent participants completed the Symptom Checklist-90-R (SCL-90-R; [1,2] and the Funf-Faktoren-Fragebogen fur Kinder, (FFFK; [17,18]).

### 3.3. The Symptom Checklist-90-R (SCL-90-R [1,2])

Given the Greek speaking population, the Greek adaptation of the SCL-90-R was used in this study. Psychometrically, the SCL-90-R has been demonstrated to possess acceptable internal consistency, retest reliability, and validity (e.g., [19,20,21]). The SCL-90-R was described in some detail in the introduction. Each item in the SCL-90-R is rated on a 5-point Likert scale, ranging from 0 (“not at all”) to 4 (“very much”), with higher scores indicating a greater severity of the symptom experienced in the last seven days. An example of an item is “Headaches”, which is part of the somatization scale. For this study, the dimension scores were used as observable indicators in the Confirmatory Factor Analysis (CFA). The internal consistency (Cronbach’s alpha) values for the subscales in this study were as follows: somatization = 0.856, obsessive–compulsive = 0.779, interpersonal sensitivity = 0.809, depression = 0.853, anxiety = 0.840, hostility = 0.823, phobic anxiety = 0.723, paranoid ideation = 0.738, and psychoticism = 0.765.

### 3.4. Funf-Faktoren-Fragebogen fur Kinder, FFFK [17,18]

The Greek version of the Five Factor Questionnaire for Children, known as the Funf-Faktoren-Fragebogen fur Kinder (FFFK; [17,18]), was used to measure the Big-Five personality traits of conscientiousness, emotional stability, extraversion, agreeableness, and openness. The FFFK consists of a series of bipolar adjective pairs, balanced for positive and negative stems in the first position [17]. Each personality dimension is represented by eight adjective pairs, with each pair rated on a five-point Likert scale (i.e., always, most of the time, neutral, rarely, and never), anchored by its adjectives. The internal consistency (Cronbach’s alpha) values for the extraversion, emotional stability, conscientiousness, and agreeableness dimensions were 0.73, 0.65, 0.61, and 0.73, respectively. The internal consistency for openness was low at 0.37.

### 3.5. Procedure

This current study was conducted in Athens, Greece, with data collected between 2010 and 2012. Approval was obtained from the research ethics committee of the pedagogical institute of the Ministry of Education (protocol number 101359Γ2). Participants were preselected randomly by lottery based on the location and type of school (academic vs. vocational track schools) to ensure an inclusive and representative sample. Additionally, no exclusion criteria were used as the aim was to collect an inclusive and representative sample. All students attending a public Greek high school were eligible to participate in this study, including those from after hours, vocational, and public high schools. The presence or absence of psychopathological symptoms was not assessed prior to this study; thus, students who likely presented with potential psychopathology were included.

Following this, parental or guardian consent was sought and obtained for all participants, with a response rate exceeding 95%. The estimated maximum sampling error was 2.70% at the 95% level of confidence (z > 1.96). The final dataset comprised 1594 respondents with complete parental consent and the full set of ratings required for the study. Data collection was conducted by a specially trained research team of 13 undergraduates, postgraduates, and PhD students of psychology. The data were collected in the participants’ classrooms during the first two or the last two school hours of a school day, in accordance with the permission provided by the Ministry of Education. The data collection process took approximately 45 min. Participation was anonymous and voluntary, with no incentives offered, and participants were not penalized for withdrawing or discontinuing their participation.

### 3.6. Statistical Analysis

M*plus* Version 7 [22] was utilized to analyze the two HiTop-based SCL-90-R CFA structural models, employing MLR extraction. At the statistical level, the global fit of the models was examined using the chi-square test. However, due to the inflation of chi-square statistic with large sample sizes, several approximate fit indices have been proposed. The approximate fit indices provided in M*Plus* include the root mean square error of approximation (RMSEA), Tucker–Lewis Index (TLI), comparative fit index (CFI), and standardized root mean square residual (SRMR). According to Hu and Bentler [23], a two-index approach to evaluating model fit is recommended, focusing on good fit in terms of the SRMR value and either the TLI, CFI, or RMSEA. For the current study, a globally good fitting model was defined a priori using these recommendations. The guidelines proposed by Hu and Bentler [23] indicates that RMSEA ≤ 0.06, CFI and TLI ≥ 0.95, and SRMR ≤ 0.08 signify good model fit. Additionally, RMSEA values between 0.06 and 0.08, CFI between 0.90 and 0.95, and SRMR between 0.08 and 0.10 indicate adequate model fit.

In Model 1, the latent factors for antagonistic disinhibited and somatoform had only one indicator each, and in Model 2, the latent factors for antagonistic disinhibited had only one indicator. When there is only one indicator per latent factor, the model must be specified with the unstandardized loading for the indicators set to 1 and the error variance fixed to a theoretically meaningful value, corresponding to the best estimate of the error variance in the indicator. In this study, the error variance for such indicators was derived from the internal consistency alpha reliability for the relevant dimensions based on their items. According to the classical test theory, the error variance for an observed variable (indicator) Y can be computed as Var (error) = Var (Y) × (1 − reliability). Using this formula, the error variances for the SCL-90-R hostility variable in the antagonistic disinhibited spectrum and the SCL-90-R somatization variable in the somatoform spectrum were determined to be 0.146 and 0.058, respectively.

In addition to acceptable global fit, model acceptance in this study required the loadings of the indicators in the model to be significant and salient (>0.30; [24]) and for the factors to demonstrate acceptable discriminant validity (*r* < 0.85; [25]) and reliability [26]. For alpha coefficient reliability, guidelines for acceptability have ranged from 0.70 and above [26]. In the current study, values of at least 0.70 were considered acceptable. For the internalizing spectra, which had five dimensions, the alpha coefficients were computed using the total scores for the five dimensions, resulting in an alpha coefficient value of 0.900. For the antagonistic disinhibited and somatoform dimensions, which each had only one indicator, internal consistency was computed using their relevant items scores (i.e., hostility and somatization), resulting in a value of 0.841 and 0.856, respectively.

To examine external validity, the unique associations between the factors in the preferred model and the Big-Five personality dimensions were assessed by regressing the latent factors on the Big-Five personality dimensions. Support for external validity was assumed if the relevant SCL-90-R-based spectra of the SCL-90-R were associated with the relevant Big-Five personality dimension, as speculated (i.e., internalizing spectrum negatively with emotional stability; antagonistic disinhibited spectrum negatively with agreeableness; paranoid ideation and psychoticism negatively with agreeableness). Given that this involved computing 15 path coefficients, the alpha for establishing significance was adjusted to *p* = 0.003 (0.05/15) to control for Type I error.

Soper’s [27] software for computing sample size requirements for CFA models was used to evaluate the sample size required for this study. For both Models 1 and 2, the anticipated effect size was set at 0.3, power at 0.8, and probability at 0.05. For Model 1, the number of latent variables was set at three, and the number of observed variables at seven. For Model 2, the number of latent variables was set at four, and the number of observed variables at nine.

## 4. Results

### 4.1. Sample Size Requirements

The analysis for sample size requirements relevant to Model 1 recommended a minimum sample size of 323, and the analysis relevant to Model 2 recommended a minimum sample size of 341. Therefore, with 1594 participants in this study, our sample size was more than adequate for testing both Models 1 and 2.

### 4.2. Examining Global Fit and Acceptability of Models Tested

The fit values for the two models tested in the study are shown in Table 1. Both models showed poor fit in terms of their RMSEA values but adequate fit in terms of their CFI and TLI values. For both models, the SRMR values indicated a good fit. Although the RMSEA did not show good fit, this need not be a source of concern. A simulation study by Kenny et al. [28] showed that models with few *df* (applicable to the current study; see Kenny et al. [28] where they show poor RMSEA values even when they are good fitting. Indeed, they have argued that using the RMSEA to assess the model fit in models with small *df* is problematic and potentially misleading and have urge researchers, reviewers, and editors not to dismiss models with large RMSEA values with small *df* [28]. Thus, our findings can be interpreted as providing an adequate fit for both models. Figure 1 shows the factor loadings and correlation between the factors in Models 1 and 2. For both models, all items loaded saliently and significantly on their respective factors, clearly defining the factors in both models. As shown in Figure 1, for Model 2, the correlations of all the latent factors, except that between internalizing and detachment, were all less than 0.85. The correlation between internalizing and detachment was 0.99, indicating no support between these factors in this model. For Model 1, the correlations of all the latent factors were less than 0.85, indicating support for the discriminant validity between all the factors in his model. When considered together, these findings suggest that Model 1 is the better and preferred model.

### 4.3. Examining Reliabilities of the Factors in Model 1

For the internalizing spectra, which comprised five dimensions, the alpha coefficient value based on the total scores for these dimensions was 0.90. For the antagonistic disinhibited and somatoform factors, which had only one indicator, the internal consistency values, based on their relevant items scores (i.e., hostility and somatization, respectively), were 0.841 and 0.856, respectively. As these values exceed the threshold for meaningful interpretation of a factor (i.e., α values > 0.70; [26]), the reliabilities of all three factors in this model can be interpreted as adequate.

### 4.4. Examining the Unique Associations of the Factors in Models 1 with Big-Five Personality Dimensions

Table 2 shows the predictions of all the factors in Model 1 by the Big-Five personality dimensions. As shown, somatization was predicted negatively and significantly by emotional stability (shared variance = 12.89%), and internalizing was also predicted negatively and significantly by emotional stability (shared variance = 27.35%). Antagonistic disinhibited was predicted negatively and significantly by emotional stability (shared variance = 12.39%) and agreeableness (shared variance = 9.42%). Overall, these finding align with the predictions of the HiTop model.

## 5. Discussion

This study examined whether and how the SCL-90-R can be effectively utilized as a measure for the HiTop model. It evaluated two different structural models in which the SCL-90-R dimensions were grouped into the various spectra of the HiTop model. In one model (referred to as Model 1), only seven of the nine SCL-90-R dimensions were included (excluding paranoid ideation and psychoticism dimensions). In this model, SCL-90-R somatization was located in the somatoform spectrum; obsessive–compulsive, interpersonal sensitivity, depression, anxiety, and phobic anxiety dimensions were located in the internalizing spectrum; and hostility was located in the antagonistic disinhibited spectrum. In the other model (referred to as Model 2), all nine SCL-90-R dimensions were included, with an additional detachment spectrum encompassing paranoid ideation and psychoticism. The support for these models was examined in terms of global fit, salience and significant of the factor loadings, discriminant validity of the latent factors, internal consistency reliability coefficients of the latent factors, and external validity of the latent factors in terms of their associations with the Big-Five personality dimensions as would be predicted by the HiTop model.

The findings indicated adequate global fit for both models, with salient and significant factor loadings. While all the factors in Model 1 showed discriminant validity, there was no support for the discriminant validity between the internalizing and detachment spectra in Model 2. The three factors in Model 1 also showed adequate reliability. Furthermore, the somatization spectrum and the internalizing spectrum in Model 1 were predicted negatively and significantly by emotional stability, and the antagonistic disinhibited spectrum was predicted negatively and significantly by emotional stability and agreeableness. Except for the prediction of the antagonistic disinhibited spectrum by agreeableness, these predictions aligned with the expectations of the HiTop model. Therefore, these findings can be taken as supporting Model 1 over Model 2. As previously discussed, Model 1 includes seven of the nine SCL-90-R dimensions with somatization located in the somatoform spectrum; obsessive–compulsive, interpersonal sensitivity, depression, anxiety, and phobic anxiety dimensions located in the internalizing spectrum; and hostility located in the antagonistic disinhibited spectrum. This support aligns with the findings of Gomez et al. [3] on the use of SCL-90-R for the HiTop model. Our current and past studies indicate that the SCL-90-R scales of somatization, obsessive–compulsive, interpersonal sensitivity, depression, anxiety, hostility, and phobic anxiety can be used successfully as measures for the HiTop subfactors/dimensions with the same names. In contrast, the SCL-90-R scales for paranoid ideation and psychoticism are not appropriate for this purpose.

While this study provides valuable insights into the utilization of the SCL-90-R for evaluating the HiTOP model, several limitations must be considered. First, background factors such as age, gender, and ethnicity, which could influence SCL-90-R items rating, were not controlled, potentially confounding the findings. Second, the impact of non-respondents or those omitted due to missing values on the results is unknown. Third, the community sample used was not truly random, limiting generalizability, and may not apply to clinically diagnosed adolescents. Fourth, the self-report nature of the SCL-90-R and the FFFK questionnaires may have introduced the common method variance effect. Fifth, the lack of exclusion criteria meant that participants with various conditions (e.g., medication use, drug addiction, intellectual disability, psychological problems, and literacy issues) could have provided confounding data. Sixth, thid study indirectly examined the alignment of the SCL-90-R dimensions with the HiTOP dimensions via the FFM, not directly in terms of how the SCL-90-R dimensions were associated with HiTOP dimensions. However, given the data collection predates the first articulation of the HiTOP model [4], no HiTop-specific measures were available nor included.

In conclusion, the findings support the use of the SCL-90-R in clinical practice and in research related to the HiTop model. More broadly, our study provides a methodological approach for identifying existing measures suitable for assessing psychological disorders from a HiTop perspective, encouraging further research in this area while considering the methodologies and limitations highlighted. We hope that the findings and interpretations in the present study, along with those from previous studies [3], will be considered by the HiTop Clinical Translations workgroup as they continue to search for existing measures to assess psychological disorders from a HiTop perspective [9,29]. This is particularly relevant for the SCL-90-R scales for somatization, obsessive–compulsive, interpersonal sensitivity, depression, hostility, phobic anxiety, and anxiety.

## Figures and Tables

**Table 1:** Fit of the HiTop-based SCL-90-R spectra models.

Model	*df*	χ^2^	RMSEA (90% CI)	CFI	TLI	SRMR
Model 1	13	333.482	0.124 (0.113, 0.136)	0.939	0.901	0.035
Model 2	23	491.653	0.113 (0.104, 0.122)	0.938	0.904	0.034

Note. χ^2^ = maximum likelihood χ^2^, RMSEA = root mean square error of approximation; CFI = comparative fit index; TLI = Tucker–Lewis index; SRMR = standardized root mean square residual. All χ^2^ values were significant (*p* < 0.01). Model 1 that excluded the SCL-90-R paranoid ideation and psychoticism dimensions had latent factors for internalizing, antagonistic disinhibited, and somatoform. Model 2 that included the SCL-90-R paranoid ideation and psychoticism dimensions had latent factors for internalizing, antagonistic disinhibited, somatoform, and detachment.

**Table 2:** Standardized path coefficients for the predictions of the latent factors in Model 1 by the Big-Five personality dimensions.

	Somatoform	Internalizing	Antagonistic Disinhibited
Extraversion	0.007	−0.073 *	0.083 **
Emotional stability	−0.359 ***	−0.523 ***	−0.352 ***
Agreeableness	−0.023	0.015	−0.307 ***
Conscientiousness	0.013	0.065 *	−0.064 *
Openness to experience	−0.044	−0.030	0.112 **

Note. *** *p* < 0.001; ** *p* < 0.01; * *p* < 0.05.

## Data Availability

Dataset not published due to privacy and/or ethical restrictions. Dataset can be made available upon reasonable request.

## Supplementary Materials

The following supporting information can be downloaded at: https://www.mdpi.com/article/10.3390/pediatric16040093/s1, Table S1: Proposed Mapping of the SCL-90-R Dimensions in the HiTOP Model and the FFM Model: An Update.
