# Supplementary material for: A Re-Evaluation of the Utility of Symptom Checklist-90-Revised for Measuring the Spectra in the Hierarchical Taxonomy of Psychopathology
Source: Pediatr Rep. 2024 Dec 9;16(4):1089–98. doi: 10.3390/pediatric16040093 (PMC11678436; doi:10.3390/pediatric16040093)
Supplement: Supplementary file 1 [file pediatrrep-16-00093-s001.zip › pediatrrep-3305532-supplementary.pdf]

Supplementary Table S1

Proposed Mapping of the SCL-90-R Dimensions in the HiTOP Model and the FFM Model: An Update

SCL-90-R Dimensions		HiTOP Hierarchy			FFM
Scale/Dimensions	Major areas covered	Subfactor	Spectra	Superspectra	Personality
Somatization	Cardiovascular, gastrointestinal, respiratory, and other systems involving autonomic mediation.	Not specified	Somatoform	Emotional Dysfunction (Somatoform)	Low emotional stability
Depression	Dysphoric mood, withdrawal, low motivation/energy, hopelessness, suicidal thoughts, somatic correlates of depression	Distress	Internalizing	Emotional Dysfunction	Low emotional stability
Anxiety	Nervousness, tension, trembling, feelings of terror and panic, somatic correlates of anxiety	Distress	Internalizing	Emotional Dysfunction	Low emotional stability

Obsessive-compulsive	Irresistible thoughts, impulses, and actions that are experienced as unwanted nature	Fear	+Internalizing	Emotional Dysfunction	Low emotional stability
Phobic anxiety	Persistent, irrational & disproportionate fear of a specific person, place, object	Fear	+Internalizing	Emotional Dysfunction	Low emotional stability
Paranoid ideation	Projective thinking, hostility, suspiciousness, grandiosity, fear of loss of autonomy, delusions	Thought	Detachment (Thought disorder)	Psychosis	High openness
Psychoticism	Withdrawal, isolation, schizoid lifestyle, schizophrenia symptoms	Thought	Detachment (Thought disorder)	Psychosis	High openness
Hostility	Aggression, irritability, rage, resentment	Antisocial	Antagonistic Disinhibited	Externalizing	Low agreeableness

Interpersonal sensitivity	Self-deprecation, uneasiness, and discomfort during interpersonal interactions	Not specified	Detachment (internal)	Psychosis	Low emotional stability
